# Demystifying “Hyaline Angiopathy” of Pulse Granuloma in Oral and Extraoral Surgical Pathology

**DOI:** 10.1111/odi.15287

**Published:** 2025-02-17

**Authors:** Felipe Fornias Sperandio, Matheus de Castro Costa, Marina Lara de Carli, Rani Kanthan

**Affiliations:** ^1^ Oral Pathologist, Oral Medicine Specialist College of Dentistry, University of Saskatchewan Saskatoon Saskatchewan Canada; ^2^ Bauru School of Dentistry University of São Paulo Bauru São Paulo Brazil; ^3^ College of Dentistry Federal University of Alfenas Alfenas Minas Gerais Brazil; ^4^ Department of Pathology and Laboratory Medicine College of Medicine, University of Saskatchewan Saskatoon Canada

**Keywords:** gastrointestinal pathology, giant cell hyaline angiopathy, hyaline ring granuloma, oral pathology, pulse granuloma, see storage cell granuloma

## Abstract

**Background and Objectives:**

Pulse granuloma (PG), or giant cell hyaline angiopathy, is an immune‐mediated reaction often following the implantation of plant‐derived food particles. PGs are primarily found in the oral cavity and gastrointestinal tract and may represent a histopathological pitfall, being mistaken for other granulomatous conditions or tumors. This study is the first to compare oral to extraoral PGs, aiming to clarify the “hyaline angiopathy” seen in PGs by developing a detailed histochemical and immunohistochemical profile of oral and colonic PGs.

**Methods:**

A computer search of 135,972 surgical pathology cases was conducted. PG histopathological slides, along with demographic and clinical data, were reviewed. Stains including Congo red, Masson trichrome, PAS, and immunostains CD31, ERG, and D2‐40 were applied.

**Results:**

Sixteen cases (11 oral, 5 extraoral) were identified, ranging from 7 to 81 years of age. Oral PGs were linked to odontogenic cysts, while extraoral PGs were associated with intestinal inflammation and perforation. Angiolymphatic marker expression was limited to the inflamed connective tissue surrounding PG.

**Conclusions:**

Our findings suggest that PGs reflect a granulomatous response to edible components and support surgical excision. The absence of vascular markers indicates that the term “angiopathy” is misleading, proposing that these “wormy” structures are fibrocollagenous responses.

## 1 Introduction

Pulse granuloma (PG) is a rare and distinctive form of foreign‐body granulomatous reaction characterized by a histological response pattern comprised of eosinophilic or hyaline material that is often associated with multinucleated giant cells and macrophages, forming granulomatous structures within affected tissues (Kimura et al. 2021). PG derives its name from the food debris it is most commonly associated with, that is, an edible seed from a legume plant known as a “pulse” (DeRoche et al. 2017; Head 1956). Also, the “giant cell hyaline angiopathy” described in PG occurs in lesions from the beginning (mouth) and ending (colonic) of the digestive tract. Most cases of PG do arise in the oral cavity, however it may also affect the lungs, in and around the gastrointestinal tract, and other sites (Rosen et al. 2022).

The clinical presentation of PG varies depending on the anatomical site. In the oral cavity, PG often presents as a localized manifestation associated with chronic inflammation and typically linked to odontogenic cysts or secondary to sites of previous dental extraction (Datar et al. 2017; Kotrashetti et al. 2011; Talacko and Radden 1988). In contrast, extraoral PG, particularly in the gastrointestinal tract, may be associated with a history of intestinal injury or disease, such as diverticular disease, perforations, fistulas, or inflammatory bowel disease (Nowacki et al. 2015). These diverse clinical scenarios highlight the importance of a thorough clinical and pathological examination to accurately diagnose PG and differentiate it from other conditions.

The pathogenesis of PG has been hypothesized to involve the introduction of foreign material, such as food particles, into the tissue, which then elicits a granulomatous inflammatory response (Nowacki et al. 2015). This is consistent with the histological findings of PG, where the eosinophilic/hyaline material typically shows positivity for periodic acid‐Schiff (PAS) and Masson's trichrome stains. These staining characteristics are indicative of the presence of complex carbohydrates and collagen, which are common in foreign body reactions.

Despite its distinctive histological features, PG may represent a histopathological pitfall and be mistaken for other granulomatous conditions or tumors (Hayat and Rumman 2022). The presence of hyaline material within PG may also mimic amyloid deposits or vascular involvement (Kimura et al. 2021; Scivetti et al. 2009). Therefore, the aim of this study is to demystify this “hyaline angiopathy” that is described in PG. A comprehensive analysis of PG by examining a series of cases diagnosed over the past 20 years is provided. In addition, a histological and immunohistochemical analysis of both oral and extraoral PG cases provides insights into their pathogenesis and clinical behavior, also better characterizing the nature of the hyaline material and vasculoinflammatory response in PG.

## 2 Materials and Methods

Pathology reports that contained the words “pulse granuloma” or “giant cell hyaline angiopathy” or “seed storage cell granuloma” or “hyaline ring granuloma” were retrieved from the Anatomic Pathology Service from the last 20 years and out of a total of 135,972 surgical signed out cases 24 cases were retrieved. Out of those 24 cases, 16 cases qualified for this study, being the respective demographic data of patients such as age, gender, as well as the anatomical site of lesions, clinical impression, radiographic findings, and final diagnosis retrieved from their respective records.

The histopathological slides (hematoxylin and eosin—HE) together with their demographic data and clinical findings were reviewed. Congo red (CR), Masson trichrome (MT), periodic acid‐Schiff (PAS), and immunostains [CD31, ERG, and D2‐40 (podoplanin)] were performed on a representative formalin‐fixed paraffin‐embedded tissue block for each case with appropriate positive and negative controls. All tissue specimens had been fixed in 10% buffered formaldehyde for at least 24 h, and routine laboratory procedures followed until paraffin embedding. Sections (4 μm thick) were obtained from the paraffin‐embedded blocks, mounted on slides, and treated with 3‐aminopropyltriethoxy‐silane (Sigma Chemical Co., St. Louis, MO, USA). Slides were then deparaffinized and hydrated. For immunohistochemistry, antigen retrieval was performed, and staining was carried out using a Roche BenchMark ULTRA PLUS (Roche, Basel, Switzerland). Primary antibodies used included anti‐CD31 (Clone JC70, mouse monoclonal, Roche), anti‐podoplanin (Clone D2‐40, mouse monoclonal), and anti‐ERG (Clone EPR3864, rabbit monoclonal, Roche). Primary incubation was followed by peroxidase blocking using 3% H_2_O_2_, incubation with biotin‐labeled anti‐mouse secondary antibody, and peroxidase‐conjugated streptavidin (Roche). Counterstaining was done with Mayer hematoxylin.

Histological, histochemical, and immunohistochemical analyses were interpreted descriptively for each slide. The histochemical and immunohistochemical staining was evaluated at different magnifications (low, medium, and high), and the stained fields were classified based on the area of the PG they represented (center, immediate periphery or surrounding tissue). Two experienced pathologists (one anatomical surgical pathologist and one oral and maxillofacial pathologist) independently reviewed each slide with consensus building for final interpretation.

## 3 Results

A total of 16 cases (11 oral and 5 extraoral) were identified. The clinical data is presented in Table 1. These lesions occurred in children and adults ranging from 7 to 81 years with a male predominance in oral cases, while the extraoral cases were all females. The majority of oral PGs were associated with an inflamed odontogenic cyst or non‐healing extraction socket. Two oral cases recurred within 5 months and 11 years after the initial diagnosis and were both related to the recurrence of the primary pathosis, that is, glandular odontogenic cyst and odontogenic keratocyst. The extraoral cases were seen in: Appendix (3); Peritoneum (1) and cecum (1) associated with inflammation and perforation.

**TABLE 1:** Clinical and histopathological aspects of oral and extraoral PG cases.

							Histochemistry/immunohistochemistry						
Case #	Oral/extraoral	Age	Sex	Site	Radiology	Lesion associated	Follow‐up	CR	MT	PAS	CD31^a^	ERG	D240
1	Oral	12	F	Right and left posterior mandible	Well‐defined radiolucency	Buccal bifurcation cyst	NR; no other biopsies	−	+	−	+	−	−
2	Oral	57	M	Left maxilla	Ill‐defined radiolucency	Odontogenic keratocyst	R of OKC and PG (11 years after)	−	+	+	+	−	−
3	Oral	41	M	Right posterior mandible	Ill‐defined radiolucency	Glandular odontogenic cyst	R of PG (5 months after); other biopsies, not related	−	+	−	−	−	−
4	Oral	60	M	Left anterior maxilla	N/A	Non‐healing extraction socket	IBD; NR; other biopsies (non‐necrotising granuloma in sigmoid, right and left colon)	−	+	+	+	−	−
5	Oral	7	M	Right posterior mandible	N/A	Buccal bifurcation cyst	NR; no other biopsies	−	+	+	+	−	−
6	Oral	19	M	Left posterior mandible	N/A	Dental follicle	NR; other biopsies, not related	−	+	+	+	−	−
7	Oral	40	M	Left anterior mandible	N/A	Chronic inflammation	NR; other biopsies, not related	−	+	−	+	−	−
8	Oral	57	M	Left anterior maxilla	N/A	Nasopalatine duct cyst	NR; other biopsies, not related	−	−	+	−	−	−
9	Oral	70	F	Right mandible	N/A	Heavily inflamed odontogenic cyst	NR; other biopsies, not related	−	+	+	+	−	−
10	Oral	66	M	Left posterior mandible	Radiolucency around #38 socket	Subaccutely inflamed granulation tissue	NR; other biopsies, not related	−	+	−	−	−	−
11	Oral	22	F	Left posterior mandible	Well circumscribed radiolucency at #38	Granulation tissue	NR; no other biopsies	−	+	−	+	−	−
12	Extraoral	9	F	Appendix	N/A	Appendicitis	NR; no other biopsies	−	+	+	+	−	−
13	Extraoral	65	F	Cecum	N/A	Residual polyp cecum with cancer	NR; other biopsies, not related	−	+	+	+	−	−
14	Extraoral	63	F	Appendix and portion of Cecum	N/A	Low‐grade appendicular mucinous neoplasm	NR; other biopsies, not related	−	+	+	+	−	−
15	Extraoral	79	F	Peritoneum	N/A	Bowel obstruction in virgin abdomen	NR; other biopsies, not related	−	+	+	+	−	−
16	Extraoral	81	F	Appendix	N/A	Appendicitis	NR; other biopsies, not related	−	+	+	+	−	−

Abbreviations: CR = Congo red, IBD = inflammatory bowel disease, MT = Masson's trichrome, N/A = not applicable, NR = no recurrence after at least 2 years of follow‐up, PAS = periodic acid‐Schiff, R = recurrence.

^a^ CD31 immunostaining refers to the tissue surrounding PG.

**FIGURE 1:**
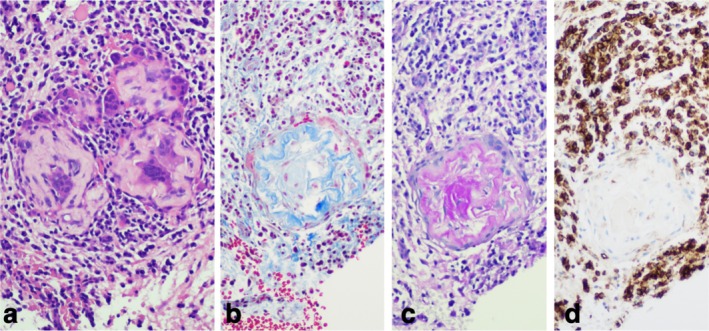
Histochemical and immunohistochemical staining profile of PG: (a) Hematoxylin and eosin staining showing wormy hyaline structures of PG amongst multinucleated giant cells. (b) Masson's trichrome (MT) staining highlighting collagen fibers within PG. (c) Periodic acid‐Schiff (PAS) positive staining in PG. (d) CD31 negative immunostaining in PG; however, positive immunostaining in the surrounding tissue.

Histologically, PG was characterized by small concentric collections of a cerebriform/wormy hyalinized material intimately circumscribed by histiocytes and multinucleated giant cells; these joint circular structures were surrounded by a primarily lymphoplasmacytic inflammatory infiltrate (Figure 1a); Russell bodies and red blood cell extravasation were often seen in the surrounding areas of PG as well. The wormy ‘hyaline angiopathy’ expressed MT: 100% of the cases stained positive; the central areas of PG showed blue staining compatible with collagen fibers, whereas the outlining periphery of PG was highlighted with a fine hyalinized pink coloration that corresponded to the cytoplasm of encircling histiocytes/multinucleated giant cells (Figure 1b). The central part of PG structures was also strongly positive for PAS; the PAS‐positive central component corresponded exactly to the MT‐positive blue staining (Figure 1c). CR was negative in all cases. There was no expression of the angiolymphatic markers ERG, D2‐40, or CD31, with the latter outlining the surrounding vasculoinflammatory response of the pulse granuloma (Figure 1d). No differences were seen in the histochemical or immunohistochemical profile of oral and extraoral cases.

## 4 Discussion

PG has been widely characterized in the literature as a type of foreign‐body granulomatous reaction/histological response pattern that is marked by eosinophilic or hyaline material often associated with multinucleated giant cells and macrophages (Kimura et al. 2021). Consistent with this characterization, PG's eosinophilic/hyaline material typically demonstrates positivity for PAS and Masson's trichrome stains (Acharya et al. 2015; Datar et al. 2017; Henriques et al. 2013; Kimura et al. 2021; Martin‐Hernan et al. 2019; Patil et al. 2017), and it is generally negative for CD34 (Henriques et al. 2013; Kimura et al. 2021; Scivetti et al. 2009).

Our study aligns with these established findings in several aspects. Specifically, we observed that the eosinophilic/hyaline material within the PGs in our cases was constantly positive for PAS and MT, consistent with most reported studies (Acharya et al. 2015; Datar et al. 2017; Henriques et al. 2013; Kimura et al. 2021; Martin‐Hernan et al. 2019; Patil et al. 2017). This confirms the histopathological consistency of PG's hallmark staining features. However, there are notable additions in our results compared to the published literature. Our findings revealed that all cases tested negative for Congo red staining, which is used to identify amyloid deposits. This result is consistent with the general understanding that PG does not feature amyloid (Nowacki et al. 2015), but could be interestingly contrasted with reports showing true Congo red‐positive eosinophilic hyaline‐rich deposits within certain granulomas and their periphery in tuberculosis patients (Ghosh et al. 2022).

A key finding of this study is the dual positivity for PAS and MT in both oral and extraoral PGs, providing insight into their structural composition. The PAS and MT staining patterns described herein are typically seen in tissues containing glycoproteins, mucins, and collagen, or structures like basement membranes, such as those found in kidneys, lungs, and intestines. Notably, the epithelial basement membranes of the oral mucosa can also be PAS‐positive due to glycoproteins like laminin and collagen IV (Juneja et al. 2006; Pujar et al. 2015). In fact, PAS specifically stains carbohydrates and glycoproteins, while MT highlights collagen fibers in blue/green (Vasanthi et al. 2022). These findings offer a better understanding of PG's composition, potentially revealing structural elements that align with the characteristics of these other well‐studied tissues.

Furthermore, our study found that the “wormy” hyaline angiopathy within the PG did not express angiolymphatic markers such as ERG, D2‐40, or CD31. Remarkably, CD31, which outlines endothelial cells and vasculature, was observed in the surrounding vasculoinflammatory response of PG in our cases, rather than within the granuloma itself. This differentiation is crucial, as it suggests that while PG itself lacks certain angiolymphatic markers, the adjacent inflammatory environment displays these markers, highlighting a distinct histological feature of PG compared to other vascular or lymphatic lesions.

In that way, this study helps us elucidate that an angiopathy is not truly present in PG and most likely does not participate in its etiopathogenesis. In fact, when the term angiopathy is used, it may lead to misinterpretation, as it usually relates to forms of vasculitis or angiitis, generally linked to rare conditions known to cause damage to blood vessels by causing inflammation or swelling, and are classified depending on the size and type of blood vessel they affect; that is, giant cell arteritis (Rebello and Joshi 2022) (affects large vessels), polyarteritis nodosa (Hernandez‐Rodriguez et al. 2014) (affects medium vessels), granulomatosis with polyangiitis (Fillingim et al. 2023) (previously known as Wegener granulomatosis (Rahilly and Rahilly 2000); affects small vessels), and Behçet disease (Mumcu et al. 2006) (affects variable‐sized vessels).

Notably, the patient's quality of life is usually significantly affected by any of these forms of vasculitis. In Behçet disease, for instance, patients will typically present with recurrent oral ulcerations plus at least two of the following clinical manifestations: recurrent genital ulcerations, eye lesions, skin lesions, and positive pathergy test. As a consequence, both oral and general quality of life are impaired in Behçet disease (Mumcu et al. 2006). Interestingly, besides not finding any signs of angiopathy in the tissue biopsies analyzed herein, the included patients did not present with any known systemic findings or any additional tissue biopsies that could indicate involvement by some sort of granulomatous or vasculitic‐type disease at the time of PG diagnosis or up to 1 year of follow‐up.

It is very important to make a distinction between PG and true angiopathic pathoses as the former can simply be treated by simple excision of the local inflammatory reaction, whereas the latter would usually require significantly more extensive treatment, usually involving systemic immunomodulating agents (Escudier et al. 2006). Given that this study is, to the best of our knowledge, the first to compare oral and extraoral PG, we are confident that our results confirm that distinction. Accordingly, PG has been successfully treated in all cases presented herein. Two recurrences were reported; however, the PG recurrence was associated with the recurrence of the primary pathosis, that is, glandular odontogenic cyst and odontogenic keratocyst; this raises a question of whether certain patients are predisposed to developing PG reactions in the presence of an inciting factor.

By comparing oral and extraoral PG cases, this study demonstrates they share great similarities from a histopathological, histochemical and immunohistochemical perspective. In that way, oral and colonic PG formation appear to reflect an immune response that is triggered by the presence of certain components in legumes/food in some individuals, and that is consistent with food, in agreement with the pertinent literature (Nowacki et al. 2015). The consistent presence of Masson trichrome and lack of vascular markers positivity within the granuloma suggest that the term “angiopathy” is not an appropriate descriptor for these lesions, and we support the recommendation that this misnomer is replaced by the previously suggested term “seed storage cell granuloma” to more precisely reflect its etiology.

The clinical presentations of extraoral PG found with this study align with those reported in the literature, which may involve a history of various intestinal injuries, including abdominal trauma, diverticular disease, perforations, and fistulas. Other relevant medical histories may include adenocarcinoma, inflammatory bowel disease, appendicitis, complications at an anastomotic site, or stent leakage (Nowacki et al. 2015). Oral lesions, on the other hand, were mostly associated with odontogenic cysts or non‐healing extraction sockets. In fact, oral PG has been found in association with post‐extraction tissue reactions (Talacko and Radden 1988) and different kinds of odontogenic cysts and tumors, such as odontogenic keratocyst (Kotrashetti et al. 2011) and ameloblastoma (Datar et al. 2017).

Although a reactive etiopathogenesis for PGs is consistently supported in the literature (often linked to the presence of certain food components or plant materials in susceptible individuals) PGs have also been found in association with entirely intraosseous lesions, including odontogenic cysts like the odontogenic keratocyst (Kotrashetti et al. 2011) and ameloblastoma (Datar et al. 2017). The exact pathophysiology of PGs in these cases remains poorly understood and has not been fully explored in the literature. Additionally, we would like to emphasize that the odontogenic and non‐odontogenic cysts that were seen in association with PGs in our study had not undergone prior surgical interventions, such as decompression or marsupialization. While our results confirm many aspects of PG's histopathological profile, they also highlight some particularities regarding Congo red staining and the expression of angiolymphatic markers. This study contributes to a more nuanced understanding of PG's histopathological features and their differential diagnosis and supports the etiopathogenesis of oral and gastrointestinal PGs to reflect a granulomatous response/histological response pattern triggered by the presence of certain components in legumes/seeds. The consistent positivity for MT with total lack of vascular immunostaining within these “wormy” structures proves there is no evidence of any ‘angiopathy’ in these granulomas. We propose these “wormy” structures represent a varied fibrocollagenous tissue response dependent on the legume/seed present, and continued usage of this term is to be strongly discouraged; for instance, “hyaline ring granuloma” or “seed storage granuloma” are terms that better describe PG (Kimura et al. 2021; Rosen et al. 2022) and are endorsed by this study.

In summary, this study helps clarify the distinction between PG and true angiopathic conditions, which may require different therapeutic approaches (Escudier et al. 2006). Understanding the exact nature of the granulomatous response in PG could lead to more precise diagnostic criteria and treatment protocols, ultimately improving patient outcomes. This research also seeks to contribute to the ongoing debate regarding the appropriate nomenclature for PG, advocating for terminology that accurately reflects the lesion's etiology and histopathological features.

## Author Contributions

**Felipe Fornias Sperandio:** conceptualization, investigation, methodology, validation, writing – review and editing, formal analysis, data curation, supervision. **Matheus de Castro Costa:** writing – original draft, visualization. **Marina Lara de Carli:** writing – original draft, visualization, validation. **Rani Kanthan:** conceptualization, investigation, writing – review and editing, validation, methodology, formal analysis.

## Conflicts of Interest

The authors declare no conflicts of interest.

## Data Availability

The data that support the findings of this study are available on request from the corresponding author. The data are not publicly available due to privacy or ethical restrictions.
